# Intervention research to protect human health in the era of climate extremes

**DOI:** 10.1371/journal.pmed.1004918

**Published:** 2026-01-29

**Authors:** Till Bärnighausen, Helen Lumbard

**Affiliations:** 1 Heidelberg Institute of Global Health, University of Heidelberg, Heidelberg, Germany; 2 Public Library of Science, Cambridge, United Kingdom

## Abstract

In this Editorial, PLOS Medicine Editor-in-Chief Till Bärnighausen and Executive Editor Helen Lumbard outline how climate change and associated extreme weather events are threatening human health, and call for research on the effectiveness of climate adaptation interventions.

## Teaser

Climate change is accelerating the frequency and severity of extreme weather events and increasingly threatening human health and life, particularly in low- and middle-income countries. Research on the effectiveness of climate adaptation interventions for human health, as well as their desirability, implementation, and financial viability, are urgently required.

## Introduction

Global climate change is accelerating the frequency and severity of extreme weather events—including heat waves, wildfires, droughts, floods, and storms—at an unprecedented pace [1], and these now-common events are acutely threatening human health and life [2]. Without successful mitigation or large-scale adaptation, extreme weather events are projected to cause nearly 15 million deaths by 2050, according to one estimate [3]. Decisions about how to protect populations are therefore not theoretical: They are being made now, often in the absence of robust evidence.

The health impacts of climate change are especially severe in low- and middle-income countries (LMICs), where most of the world’s climate-vulnerable communities are located [1]. For instance, in parts of Burkina Faso, one of the resource-poorest countries in the world, extreme temperatures above 40 °C are now observed for around 100 days every year [4], which likely leads to substantial climate-sensitive mortality [5]. Such conditions place acute strain on human physiology, livelihoods, and health systems, underscoring the urgent need for scalable and effective climate adaptation interventions that protect health.

Climate vulnerability is typically compounded by structural disadvantage. Where climates are most extreme, the infrastructure to adapt to extreme weather events is often lacking. For instance, in the Sahel, one of the world regions hardest hit by extreme heat, more than two thirds of households lack electricity (which could be used for air conditioning to protect from heat) [6], more than half lack running water (which could be used to lower body temperature) [7], and many lack safe water and sanitation that can withstand floods and droughts [8].

Climate-vulnerable communities also often lack strong health systems that could successfully treat the health risks and diseases of extreme weather events, such as heat strokes during heat waves, fractures during storms, and burns and respiratory distress during wildfires. As a result, mortality rates from extreme weather events are dramatically higher in climate-vulnerable communities than in less vulnerable ones—15 times higher, as reported by the *Intergovernmental Panel on Climate Change* [9].

## A large portfolio of interventions—But little causal evidence

Despite the scale and urgency of the problem, the evidence base on the effectiveness of climate adaptation interventions for human health remains strikingly thin. A 2021 synthesis of all climate adaptation research found a total of 1682 articles, of which 510 described “implemented human adaptation” for “health, well-being and communities” [10]. A follow-on review of this evidence identified 99 articles that reported on adaptation interventions (for extreme heat, drought, rainfall, and flooding) to specifically protect human health in LMICs [11]. These studies demonstrated engineering and implementation feasibility for many plausibly effective interventions. However, only two studies reported on “formal *ex ante* planned and designed evaluations of adaptation responses (with a counterfactual)”, i.e., results from a strong, causal evaluation of effectiveness [11]. The authors thus concluded that “there is an urgent need for significantly greater consideration of the health outcomes in evaluations of climate change adaptation responses, including pre-specified and well- designed formal evaluations.” We agree. Results on the causal effectiveness of climate adaptations for human health are urgently needed to guide policy recommendations and motivate funding and scale-up.

At *PLOS Medicine*, we are keen to receive articles that move the field from plausibility to proof. This includes randomized controlled trials of climate adaptation interventions with clearly defined health outcomes, as well as strong quasi-experiments that establish the causal effects of climate adaptation interventions on human health using modern causal inference—e.g., regression discontinuity, instrumental variable, or two-way fixed-effects designs [12] for those climate adaptation policies that cannot be randomized and prospectively studied.

Beyond health effects alone, evidence is also needed on the social desirability, economic value and financial viability of climate adaptation interventions. Decisions about scale-up require understanding not only whether an intervention works, but for whom, at what cost, and under what conditions. We therefore welcome rigorous research on desirability, acceptability, economic value and equity implications grounded in empirical measurements of implementation and costs across diverse settings [11,12].

## Priority areas for research

Effectively protecting populations from climate-related health risks will require a portfolio of complementary interventions that operate across behavioral, environmental, public health, and policy domains. Priority areas where evidence on effectiveness, desirability, and value is urgently needed include:

- Behavioral interventions—such as climate-sensitive health counseling in primary care, wildfire training for healthcare professionals, community training programs for extreme weather preparedness; and interventions boosting social connection to reduce extreme climate vulnerability [13].
- Physical interventions—such as retrofitting homes, schools, and shopping malls with cool roofs to protect humans against extreme heat; passive ventilation to reduce humidity in healthcare facilities; stormwater and rainwater capture to protect water supplies during droughts and replenish aquifers; elevation of healthcare facilities in flood-prone areas; stormwater ponds and rain gardens to protect communities against floods; storm drains to reduce the risk of water- and vector-borne diseases; and green and blue spaces to absorb heat and water [14].
- Public health programs—such as early-warning and anticipatory-action systems to help people prepare for extreme weather events and climate-informed vector surveillance and control programs.
- Health policy and regulatory innovations—such as novel occupational standards for climate-vulnerable professions, building codes to reduce indoor overheating and outdoor heat islands [4], and public subsidies to boost the resilience of health-relevant supply chains against extreme weather events.

We welcome studies evaluating single interventions as well as integrated, multi-component adaptation strategies, alongside perspectives and policy analyses addressing how best to design and implement climate–health adaptation at scale.

## A call to action

National and local governments are increasingly required to develop climate change and health adaptation plans, yet often lack the evidence needed to do so effectively. Health researchers and funders therefore have a prime opportunity to close this gap. By prioritizing rigorous causal evaluation, equity-sensitive design, and policy-relevant outcomes, the research community can help ensure that climate adaptation investments translate into real and sustained health protection for the populations who need it most.

## Abbreviation:

LMICs: low- and middle-income countries
